# Cost and mortality prediction using polymerase chain reaction pathogen detection in sepsis: evidence from three observational trials

**DOI:** 10.1186/cc9294

**Published:** 2010-10-15

**Authors:** Lutz E Lehmann, Bernd Herpichboehm, Gerald J Kost, Marin H Kollef, Frank Stüber

**Affiliations:** 1 Department of Anesthesiology and Pain Therapy, University Hospital Bern, Inselspital, Freiburgstrasse, CH-3010 Bern, Switzerland; 2 Department of Health Economics (VM), Roche Diagnostics Germany GmbH, Sandhofer Str., D-68305 Mannheim, Germany; 3 Department of Pathology and Laboratory Medicine, University of California Davis Medical Center, 3453 Tupper Hall, Davis, CA 95616, USA; 4 Department of Internal Medicine, Pulmonary and Critical Care, Washington University School of Medicine, 660 S. Euclid Ave., St. Louis, MO 63110, USA

## Abstract

**Introduction:**

Delays in adequate antimicrobial treatment contribute to high cost and mortality in sepsis. Polymerase chain reaction (PCR) assays are used alongside conventional cultures to accelerate the identification of microorganisms. We analyze the impact on medical outcomes and healthcare costs if improved adequacy of antimicrobial therapy is achieved by providing immediate coverage after positive PCR reports.

**Methods:**

A mathematical prediction model describes the impact of PCR-based rapid adjustment of antimicrobial treatment. The model is applied to predict cost and medical outcomes for 221 sepsis episodes of 189 post-surgical and intensive care unit (ICU) sepsis patients with available PCR data from a prospective, observational trial of a multiplex PCR assay in five hospitals. While this trial demonstrated reduction of inadequate treatment days, data on outcomes associated with reduced inadequate initial antimicrobial treatment had to be obtained from two other, bigger, studies which involved 1,147 (thereof 316 inadequately treated) medical or surgical ICU patients. Our results are reported with the (5% to 95%) percentile ranges from Monte Carlo simulation in which the input parameters were randomly and independently varied according to their statistical characterization in the three underlying studies. The model allows predictions also for different patient groups or PCR assays.

**Results:**

A total of 13.1% of PCR tests enabled earlier adequate treatment. We predict that cost for PCR testing (300 €/test) can be fully recovered for patients above 717 € (605 € to 1,710 €) daily treatment cost. A 2.6% (2.0 to 3.2%) absolute reduction of mortality is expected. Cost per incremental survivor calculates to 11,477 € (9,321 € to 14,977 €) and incremental cost-effectiveness ratio to 3,107 € (2,523 € to 4,055 €) per quality-adjusted life-year. Generally, for ICU patients with >25% incidence of inadequate empiric antimicrobial treatment, and at least 15% with a positive blood culture, PCR represents a cost-neutral adjunct method.

**Conclusions:**

Rapid PCR identification of microorganisms has the potential to become a cost-effective component for managing sepsis. The prediction model tested with data from three observational trials should be utilized as a framework to deepen insights when integrating more complementary data associated with utilization of molecular assays in the management of sepsis.

## Introduction

Inadequate antimicrobial treatment has been identified as an important factor contributing to mortality in sepsis [1]. The rates of initial inadequate empiric antimicrobial treatment in hospitals vary and are often reported to be in the range of 15 to 30% [2-6]. Early adequate antimicrobial treatment in septic shock patients is crucial, as mortality increases by 7.6% each hour of delay after onset of hypotension [5]. However, current laboratory methods of microbiologic testing are very time consuming [7,8] and lack sensitivity [7,9,10]. Physicians therefore start, and frequently also modify [11], empiric antibiotic therapy without an identification of the relevant microorganism.

There is a growing body of literature comparing BC and PCR methods and sketching potential clinical applications [12-16]. However, none of these papers was able to quantify the expected effects. The first demonstration of how a multiplex PCR assay is able to differentially identify sepsis patients who could benefit from a predefined intervention was [17], *observing *that 36.4 (22 to 51) days of early inadequate treatment could be eliminated per 100 PCR tests performed in the ICU if the rapidly available PCR results were used to adjust treatments.

Published interventional data using the new treatment modality are still lacking. For the time being, we, therefore, must bridge an important gap by assuming that the association between *early inadequate treatment *and *elevated mortality and morbidity *as observed in other trials [3,4,18-22] is applicable to the patient cohort which can be moved from inadequate to adequate treatment via utilization of PCR results.

The goal of this paper is to synthesize available knowledge into predictions of cost and of mortality impact of PCR testing in the management of sepsis, and to provide a framework for future inclusion of more forthcoming data in this novel and clinically interesting field.

## Materials and methods

### Study design and patients

This study builds on data of a previous study [17], including all post-surgical and ICU patients from two German, one Italian, one Spanish and one US hospital of this previous study in which a multiplex PCR test (LightCycler Septi*Fast *test, Roche Diagnostics, Penzberg, Germany) was performed in parallel to the first blood culture in a sepsis episode. Approval to use these data for re-analysis in the present study was obtained. The potential impact of utilizing all PCR reported findings *to provide rapid coverage *for these microorganisms and their potential resistances (according to the local or regional resistance data for the PCR reported microorganism) was evaluated. Furthermore, this study makes use of pooled data on *outcomes associated with inadequate treatment *from two earlier trials with combined 1,147 medical or surgical ICU patients (of which 316 were inadequately treated) [3,4]. Approval to use these data for re-analysis in the present study was obtained.

Below, we describe the models built for predicting cost-effectiveness from a perspective of total healthcare cost. Note that our cost impact and mortality predictions are independent, that is, our analysis attempts to justify cost with either one of these effects.

For mortality analysis the event of non-survival is assigned to the last episode with inadequate treatment within 30 days of death, or to the last episode if all were adequately treated, so double-counting of non-survivals is avoided.

The PCR assay [23] is available as CE marked diagnostic reagent in Europe and some other countries, but at this time is not available for diagnostic use in the USA.

### Cost impact prediction

We develop a quantitative description of how PCR may trigger lower morbidity and hence lower treatment cost, which may balance the incremental laboratory cost. Parameters we use are listed in Table 1. Full cost per PCR test, *Cost_t_*, is calculated according to Additional data file 1. According to the investigated treatment algorithm, only one PCR test is used per treatment episode to optimize antimicrobial therapy. The overall cost impact can be described with Equation 1.

**Table 1:** Parameters used to calculate cost impact and cost-effectiveness

Parameter	Description	Unit	Value	Source	Used in equation
*Cost _t_*	Full cost per PCR test	€	300	Additional file 1	*1, 4, 5*,
*DG*	Days gainable on adequate treatment when utilizing PCR+ information	day	2.78 = 80.5/29	[17]^a ^	*2,3,4*
$Du_{PCR+}^{IA}$	Mean total duration of inadequate treatment (as observed in PCR+ episodes with 0.5 ≤*Du _IA _*≤ 7.5)	day	3.28 = 0.5 + DG	[17]^a ^	*2, 5*,
$F_{LOS}^{IA}$	Factor which translates days on earlier adequate treatment into outcome in terms of mean days reduced length of stay (*deltaLOS *= *LOS_Inad _*- *LOS_Ad_*)	-	1.15 = 3.763 day/3.276 day	$=\text{​}\;\frac{deltaLOS}{Du_{PCR+}^{IA}}$ calculated from [3,4,17]	*3, 4*
$In_{PCR+}^{IA}$	Incidence of inadequate treatment in the PCR+ group	-	0.397 = 29/73	Figure 1	*4, 5*
*LY _gained_*	Mean # years survival of survivors of ICU sepsis, age cohort >60 ^c^	yr	5.43 ^b ^= 12.3 * 488/1105	[26]	*6*
$M_{PCR+}^{IA}$	Mortality rate of inadequately treated PCR+ patients	-	0.414 = 12/29	Figure 1	*5*
*RR***†**	Relative risk of non-survival (@ inadequate/adequate treatment)	-	2.315	[3,4,17] pooled	*5*
*Sh _PCR+_*	Share of episodes with at least 1 PCR+ microorganism	-	0.330 = 73/221	Table 2	*4, 5*
*TAT_PCR_*	Time between PCR sampling and result reported (hours)	hr	12	[17]	*2*
*Utility _health.state_*	Health state utility after ICU sepsis	QALY/yr	0.68	[26]	*6*

^a ^Not identical with time to positive blood culture, but linked to it in about 50% of contributing data

^b ^LY_gained _(of survivors age >60, according to data from [26]); = LY_gained _(all included sepsis survivors)* ICER(all)/ICER(age >60) = 12.3 yr * 48,800 $/110,500 $ = 5.43 yr.

^c ^We use the data for those aged >60 because the mean age in our subgroup with potential survival benefit (Table 3) is 69.1 yr.

€, Euro (European currency unit); QALY, quality-adjusted life year.

(1) $$Impact=N_{epis}*(Cost_{t}-Sav_{trig})$$

*N_epis _*is the total number of episodes (and tests) in which blood cultures are complemented by PCR testing for managing early antimicrobial treatment in sepsis.

For calculating the mean savings a PCR test triggers, *Sav_trig_*, we developed Equation 3: Savings can only occur in those patients who are PCR positive (PCR+) and on inadequate ("IA") empiric treatment (the first two factors in Equation 3). The parameter DG describes the mean days gainable on early adequate treatment (Table 1, Equation 2).

(2) $$DG=(Du_{PCR+}^{IA}-\frac{TAT_{PCR}}{24})$$

The factor $F_{LOS}^{IA}$ translates days on earlier adequate treatment into outcome in terms of days of reduced length of stay (LOS). It is calculated by dividing the mean ICU-LOS reduction by the mean duration of inadequate empiric treatment, $Du_{PCR+}^{IA}$ ([3,4,17]; Table 1).

If the savings which can be realized per day of reduced stay, *Sav_d_*, are known, the resulting savings triggered per PCR test done, *Sav_trig_*, can be calculated according to:

(3) $$Sav_{trig}=Sh_{PCR+}*In_{PCR+}^{IA}*DG*F_{LOS}^{IA}*Sav_{d}$$

However, the savings which can be realized per day of reduced stay, *Sav_d_*, are highly variable. Therefore, rather than attempting to determine the overall cost impact we conducted a break-even analysis. Zero overall cost results according to Equation 1 when the savings triggered per PCR test equals the cost per PCR test, that is, when *Sav_trig_= Cost_t_*. The value for *Sav_d _*which results with that substitution in Equation 3 is defined as the break-even cost-savings per day of LOS reduction, *Sav_be _*(Equation 4). Essentially, patients with that mean daily cost or higher can receive PCR testing without incurring net cost.

(4) $$Sav_{be}=\frac{Cost_{t}}{Sh_{PCR+}*In_{PCR+}^{IA}*DG*F_{LOS}^{IA}}$$

### Mortality prediction

In the following we describe how PCR may trigger lower mortality, and quantify the related cost-effectiveness. Parameters we use are listed in Table 1. Cost per incremental survivor can be determined according to Equation 5 when dividing the incurred cost by the number of incremental survivors. For calculating the number of incremental survivors, we developed the denominator of Equation 5. With the investigated PCR based treatment algorithm, a mortality effect can only occur in the cohort of inadequately treated PCR+ patients (first three factors in the denominator of Equation 5. The mortality observed in this cohort within 30 days of discontinuing antimicrobial treatment should be reduced according to the relative risk of non-survival, *RR***†**, when comparing inadequately to adequately empirically treated cohorts [3,4]. Using this assumption, the fraction of incremental survivors is calculated by the factor ($M_{PCR+}^{IA}-\frac{M_{PCR+}^{IA}}{RR\text{†}}$) in Equation 5.

(5) $$Cost_{surv}=\frac{N_{epis}*\;Cost_{t}}{N_{epis}*Sh_{PCR+}*In_{PCR+}^{IA}*\;(M_{PCR+}^{IA}-\;\frac{M_{PCR+}^{IA}}{RR\text{†}})\;*\;\frac{DG}{Du_{PCR+}^{IA}}}$$

The last factor in the denominator, $\frac{DG}{Du_{PCR+}^{IA}}$, is always smaller than one and thus reduces the theoretical mortality effect which would result in immediate adequate treatment but cannot be fully achieved due to the time (hours) needed to obtain PCR results, *TAT_PCR_*. (see Equation 2). The linear correlation between delay of adequate treatment and outcome that we imply is supported by data from an animal model of sepsis [24] and also evidenced with human data [25]. *N _epis _*was left in Equation 5 for easier understanding, but can be canceled out.

The incremental cost effectiveness ratio (ICER) is commonly defined by Equation 6 and follows when dividing cost per incremental survivor (as determined with Equation 5) by gainable quality-adjusted life-years (QALY) per incremental survivor. *QALY *were *not *observable in our non-interventional data. See Table 1 for our data source for mean number of life-years for sepsis survivors of the applicable age group, and for the discount factor for reduced quality of life after an ICU stay for sepsis, *Utility_health-state_*.

(6) $$ICER=\frac{Cost}{QALY}=\frac{Cost_{surv}}{Lifeyears_{gained/survivor}*\;Utility_{health-state}}$$

### Statistical analysis

To characterize the uncertainty of predicted results, we re-iterated all calculations while the input parameters were randomly and independently varied using their binomial, multinomial and log-normal distributions, as deducted from the respective statistical characterizations in [3,4,17]. Results from [4] and [3] have been pooled. In each such Monte Carlo simulation 1,000 samples have been generated, each with 1,000 patients. The (5% to 95%) percentile ranges for the predicted results are reported in the manuscript. All calculations were done with SAS version 9.1.3 (SAS Institute, Cary, NC, USA). Sensitivity of results to patient cohort characteristics is explored by making use of Equations 4, 5 and 6.

## Results

### Prediction of impact on morbidity, length of ICU stay and cost

**Figure 1:**
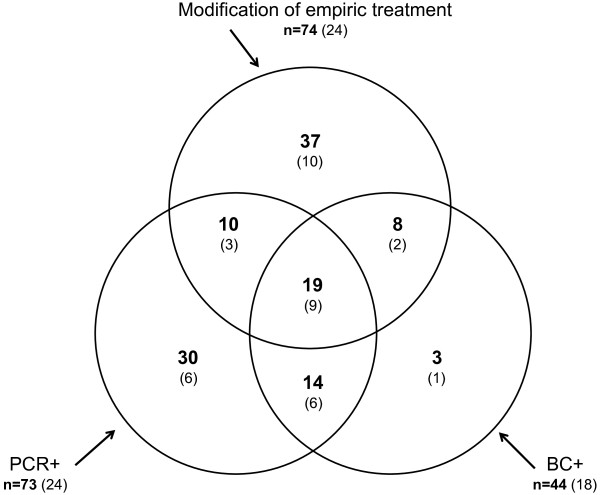
**Modification of empiric antimicrobial treatment and microbiological characterization**: Among 221 investigated sepsis episodes, 74 (33.5%) required modification of empiric antimicrobial treatment (upper circle). Positive blood cultures (lower right circle) triggered 27 (= 8 + 19) of these changes. Among 73 PCR+ episodes (lower left circle), 29 (= 10 + 19) allowed *earlier *adequate treatment (data from [17]). In brackets: Non-survivors within the respective groups.

In 74 of 221 episodes, antimicrobial treatment was modified after more than 12 hours of empiric initial treatment in order to cover different suspected or culture-determined microorganisms or resistances. PCR results (Table 2) suggested in 29 of these 74 episodes (Figure 1) equivalent antimicrobial adjustments earlier. As a consequence, 80.5 days (CI 48 to 113 days) potential earlier adequate treatment were enabled by 221 PCR tests [17], with associated costs of 66,300 €.

**Table 2:** Underlying diagnoses and identification of clinically significant microorganisms in 221 ICU or surgical ward episodes (subset of data reported earlier [17])

	Totals	PCR + episodes	**Share of PCR+ epsiodes**^**b **^ ***Sh_PCR+_***	BC+ episodes	**Share of BC+ epsiodes**^**b**^ ***Sh***_***BC+ ***_
**Total ^a^**	**221**	**73**	**0.33**	**44**	**0.20**
**Underlying diagnoses ^a^**					
- Intra-abdominal sepsis	87	31	0.36	22	0.25
- Nosocomial pneumonia	80	28	0.35	21	0.26
- Community acquired pneumonia	6	1	0.17	0	0
- Multi-organ dysfunction syndrome	7	4	0.57	4	0.57
- Catheter related sepsis; post cardiac surgery	45	18	0.40	9	0.20
- Neutropenic fever	15	5	0.33	2	0.13
- Bone/joint infection	9	4	0.44	0	0
Other	46	11	0.24	3	0.07

^a ^underlying diagnoses do not add up to totals due to dual conditions

^b ^at least one clinically significant microorganism retrieved in *one *PCR, respectively in any of *several *(as in standard of care) BC tests.

BC, blood culture (BC+: blood culture with clinically relevant microorganism identified, not counting contaminations that are immediately at reporting evident to the treating clinician); PCR, polymerase chain reaction (PCR+: PCR with clinically relevant microorganism identified. Note that in our study, *all *PCR+ reported microorganisms are considered clinically relevant.

To translate *earlier adequate treatment *into a clinical *outcome *measure, we use the factor $F_{LOS}^{IA}$ (Table 1) that predicts 1.15 days (1.02 to 1.64 days) shorter duration of ventilation and ICU stay for each day of earlier adequate treatment, or 92.5 days in total. Hence, the costs associated with PCR testing could be fully recovered in departments with mean daily treatment costs above 717 € (605 € to 1,710 €) (Equation 4 with inputs of Table 1).

Correcting inadequate coverage of a Gram-positive pathogen by multiplex PCR contributed 38.2% to the reported cost-effectiveness; the contributions of accelerated Gram-negative or antifungal coverage were 46.3% and 15.5%, respectively.

**Figure 2:**
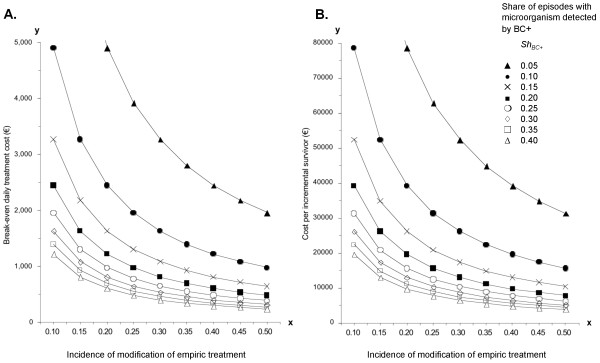
**Impact from PCR testing**. Diagrams for estimating impact from PCR testing in sepsis, based on incidence of modification of initial antimicrobial treatment (x-axis) and share of episodes with positive blood culture (curves): **A: **Cost-neutral application of PCR is predicted if the mean daily treatment cost of those included in PCR testing *exceeds *the break-even value on the y-axis (A). Data point from our study: 717 €, at 20% BC+ (Table 2) and 33.5% modification of empiric treatment (= 74/221, Figure 1). **B: **Cost per incremental survivor is predicted as indicated on the y-axis (B). Data point from our study: 11,477 €, at 20% BC+ and 33.5% modification of empiric treatment. Figure 2 was calculated using equation 4 (A) and equation 5 (B) with substitution terms as given in Additional data file 2.

Generally, for any sepsis patient cohort characterized by incidence of antimicrobial modifications (x-axis) and observed *BC+ *share (curves), Figure 2A allows to estimate which mean daily treatment cost savings would be required in order to recover the PCR related costs.

### Prediction of impact on mortality

**Figure 3:**
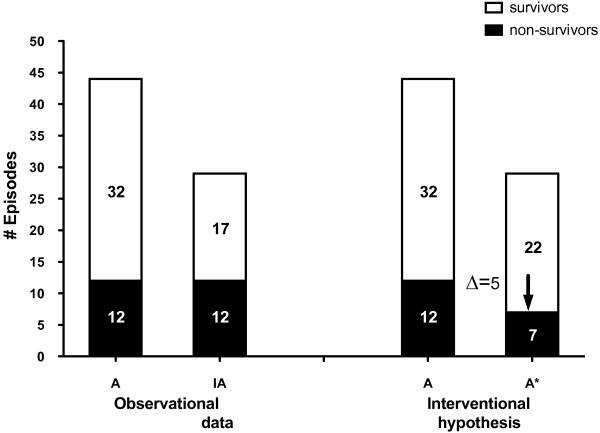
**Predicted mortality reduction**. Predicted mortality reduction if earlier adequate treatment is achieved in 29 of the 73 PCR+ episodes: **A: **Under adequate treatment, a 27.3% mortality is observed (= 12/(12 + 32)); IA: In the inadequately treated (IA) group, a mortality of 41.4% is observed (= 12/(12 + 17)); A*: A PCR+ based intervention should reduce the mortality in the former IA group according to equation 5 and the reduced relative risk of dying, *RR***† **(from [3,4]). We predict that 5 of the 12 (Table 3) non-survivors might have survived if the PCR+ results were interventionally used. The resulting mortality of 26.0% (= (12 + 7)/73) is comparable to the mortality observed under adequate treatment (A).

Thirty-day non-survival was observed in or after 26.7% (59/221) of all episodes, and 32.9% (24/73) of PCR+ episodes (Figure 1). Of 73 PCR+ episodes, 44 (thereof 12 non-survivals; Figure 3A) were adequately treated and hence without potential impact from PCR. However, in 29 inadequately treated PCR+ episodes (Figure 3, IA), another 12 non-survivors were observed without PCR based intervention (Table 3). The earlier adequate treatment facilitated by the PCR+ results translates into five lives potentially saved (Figure 3A*) if we use the relative risk of non-survival associated with initial inadequate treatment of 2.32 (CI 1.96 to 2.74; *P *< 0.001), as established previously [3,4]; furthermore, we factored in a correction because PCR+ driven adjustments are not immediate (Equation 5). Our prediction translates into an absolute reduction of mortality by 2.6% points (2.0 to 3.2%).

**Table 3:** Characteristics of 12 non-survivors observed in 29 inadequately treated PCR+ patients

Age	Co-morbidity	Infectious focus	PCR+ pathogen ^a^	# days gainable ^b^	Evidence for PCR+ relevance ^c^	
					BC+	Other test
74	Pleural lesion	Peritonitis	*Aspergillus, Candida*	4	*Candida*	*Aspergillus *antigen+
79	Decompensated heart (right side)	Cholangitis	*Pseudomonas, (Escherichia coli)*	7	*Pseudomonas*	Bile- duct cul+
66	Liver transplantation	Peritonitis	*Stenotropho-monas*	4	*Stenotropho-monas*,	Tracheal swab cul+
77	Hemodialysis	Catheter-related	CoNS ^d^	2	CoNS (2×)	Pos. tracheal swab cul+
47	Trauma	Pneumonia	CoNS ^d^	2	CoNS, *Pseudomonas*	Catheter-tip CoNS+
55	Poly-trauma	Abdominal (late detected)	*Enterobacter*	7	*Enterobacter*	*Enterobacter *in cul+
62	Cardiothoracic surgery	Pneumonia; unclear: 2^nd ^focus	*Staph.aureus: *MRSA	2	MRSA+ (3×)	Thorax, sternum cul+
58	Artherosklerosis	Pneumonia	*Aspergillus*	2.5	-	Bronchial aspirate cul+
78	Rectal neo-plasm; perforat-ed abscess	Intra-abdominal	*Enterococcus faecium*	1.5	*Enterococcus faecium* (*post mortem*)	*Enterococcus faecalis *in drainage cul+
85	Cardiac surgery	Pneumonia	*Klebsiella*	3	*Enterobacter *(equivalent the- rapy change )	
71	Bypass surgery	Pneumonia	*Enterococcus faecalis; (Pseudomonas)*	3	*Enterococcus faecalis*	*Pseudomonas *in cul+
77	Cardiac surgery	Pneumonia	*Klebsiella*	5	*Klebsiella*	*Klebsiella *in cul+

^a ^Insufficiently empirically covered PCR+ microorganisms, and (concurrent other PCR+ microorganism).

^b ^Days gainable on early adequate coverage if the PCR+ information is utilized.

^c ^*Main *evidence is the *clinical *course associated with antimicrobial treatments. In the columns below we report other *laboratory *findings that suggested drug changes equivalent to those PCR could have triggered earlier (see column #days gainable).

^d ^in PCR+: above manufacturer cut-off for CoNS.

BC+, blood culture with clinically relevant microorganism identified, not counting contaminations that are immediately at reporting evident to the treating clinician; CoNS, coagulase-negative *Staphylococcus; *cul+, microorganisms found in cultures of specimen other than positive cultures; MRSA, methicillin-resistant *Staphylococcus aureus*; PCR, polymerase chain reaction (PCR+: PCR with clinically relevant microorganism identified. Note that in our study, *all *PCR+ reported microorganisms are considered clinically relevant

With full cost associated with PCR testing of 300 €/test, the cost per incremental survivor calculates to 11,477 € (9,321 to 14,977 €) (Equation 5 with inputs of Table 1). To determine the incremental cost-effectiveness ratio (*ICER*) of 3,107 € per QALY (2,523 to 4,055 €/QALY) we used Equation 6 with data on life-years for a cohort of survivors of severe sepsis aged >60 from reference [26] (Table 1).

Figure 2B allows us to estimate the cost per incremental survivor when the PCR method is used in any severe sepsis patient cohort (with about 30% mortality) that can be characterized by incidence of antimicrobial modifications (x-axis) and observed *BC+ *share (curves).

### Potential risk of false-positive PCR results

In the post-surgical and ICU group of patients we saw a concurrent risk of unnecessary rule-in of extended antimicrobials in 6 of 221 episodes.

## Discussion

We believe that our paper (a) represents the first quantitative evaluation of expected cost and outcomes from PCR-based interventions in sepsis; (b) offers a framework to assess which patient groups might benefit most; (c) can provide valuable guidance, notably when designing and evaluating interventional trials that incorporate PCR into managing antimicrobial treatment in sepsis; and (d) will lend itself to assess relative utility and cost-effectiveness of alternative molecular diagnostics assays.

The investigated PCR assay identified patients who could benefit from a predefined intervention. Among 73 episodes with 87 positive PCR findings, in 29 the information gain was useful as there was a need to alter the antimicrobial treatment. Other authors have observed rates of inadequate coverage of similar magnitude in blood culture positive patients in ICUs [2-6,27]. However, a concurrent risk of over-treatment was observed [17]. The unnecessary rule-in of extended antimicrobials (vancomycin, oxazolidinone, piperacillin/tazobactam, a carbapenem, or an antifungal; [17]) in 6 of 221 episodes are +2.3% on top of the 260 empiric courses of extended treatment in our trial, that is, a comparatively small incremental burden of cost for antimicrobials or of dealing with incremental side-effects. While cost for antimicrobial drugs are expected to rise in the *early *treatment days, we hypothesize that overall the burden of unnecessary extended treatment will be *reduced *by utilization of PCR+ information for earlier adequate treatment. The aspect of false positive PCR assays and differential costs for antimicrobial drugs was not included the cost analysis. These secondary effects are to look for in future interventional trials.

We did not make any use of negative PCR findings. Single or consecutive negative PCR findings in certain patient types and clinical situations may be useful for early de-escalation strategies of antimicrobial treatment. However, our data show 11 cases of positive blood culture with negative PCR assay (Figure 1). Therefore, we conclude withdrawal of antimicrobial treatment upon a PCR negative result is not recommended. Furthermore, regarding the overall effect, it would be entirely possible that inadequate discontinuation after PCR negative results would cancel out the improved treatment from PCR positive findings.

Given the expected minor impact on drug cost, but high efforts required for the multiplex PCR method in the laboratory [23], significant concerns about cost-effectiveness prevail [28]. We demonstrate that improved morbidity through earlier adequate treatment leads to full recovery of PCR cost for the patients studied by us if their mean daily cost was at least 717 € (605 € to 1,710 €). In the ICU, where daily costs exceed 1,710 € [29], there is over 95% likelihood of lowered overall cost. For different patients than those included in our five study sites, Figure 2A can be used for defining whether PCR testing should be implemented. In clinical reality, the actual value of a freed-up ICU bed will vary, but it should not be underestimated: According to a recent study [30], discharge from the intensive care unit at a time of no vacancy was a significant risk factor for intensive care unit readmission or unexpected death.

Mostly, we studied ICU patients with predominantly hospital-acquired infections, not community-acquired. For the latter, the rate of initial inadequate treatment typically is lower [4]. Furthermore, the daily average treatment cost of community-acquired infections might be less than hospital-acquired. However, both inadequate rate and daily treatment cost are key determinants of cost effectiveness (see Equation 4). Equation 4 can be employed to predict cost effectiveness for community acquired infections if the input data, such as rate of inadequate treatment and average daily treatment cost are known.

Another hidden cost of inappropriate antimicrobial therapy is the increasing prevalence of *Clostridium difficile *associated diarrheal illness. Savings could be generated from lower *C. difficile *incidence with lower rate of inadequate antimicrobial treatment. This aspect is not included in our model as only interventional studies would allow quantitative observations.

Besides the cost impact, we analyzed the potential of the new method to lower mortality from sepsis. The predicted 2.6% (2.0 to 3.2%) absolute reduction of mortality in the PCR-tested patients could be considered a relevant contribution to the Surviving Sepsis Campaign [21,31]. In big patient cohorts with severe sepsis, inadequate antimicrobial treatment has been identified as independent predictor of mortality [21,32]. Specifically in septic shock, Kumar *et al. *[5] observed an increase of mortality by 7.6% each hour of delay after onset of hypotension. While the PCR method with its minimum turn-around time of 6.3 hrs [33] seems not well-suited for becoming focused on septic shock patients, shock survivors still might recover faster after the PCR+ triggered earlier treatment adjustment. Therefore we conclude that the principal use of the method should be broad and early in sepsis and severe sepsis, so the progression of disease towards septic shock might be reduced.

For the 29 post-surgical or ICU patients with potential PCR+ impact we observed a mortality of 41.4% (Figures 1 and 3). To predict the potential mortality reduction from PCR+ triggered earlier adequate treatment, we used a relative risk of non-survival between immediate and delayed adequate antimicrobial treatment (*RR***†**) of 2.32 (CI 1.96 to 2.74; *P *< 0.001). This is well within the range of observations given in literature for this relative risk, typically in the range of 1.3 to 3.8 [20-22], but even up to 10 [19]. A statistically analyzed multi-center dataset reported by Harbarth *et al. *[32], with attention paid also to isolating confounding variables, yielded a *RR***† **of 1.8; it might be a better estimate than the one we used; however, the resulting cost per incremental survivor of 14,670 € is in the magnitude of our calculated result (Figure 2B). To determine the incremental cost-effectiveness ratio (*ICER*) we used data on life-years after ICU for a cohort aged >60. If younger patients were included [26], or if following other references [34], significantly lower ICER results would be obtained (1,350 or 1,053 €/QALY instead of 3,107 €/QALY).

The cost per incremental survivor of 11,477 € (9,321 € to 14,977 €) and incremental cost-effectiveness ratio (*ICER*) of 3,107 € (2,523 € to 4,055 €) per quality-adjusted life-year that we predict are well below what has been reported for other sepsis-related strategies, notably for drotrecogin alfa (activated) [26,35,36]. In hospitals where this drug is used as rescue strategy in late Sepsis stages, utilizing PCR as adjunct should elevate the overall cost-effectiveness while probably improving the overall mortality outcome further.

A key limitation of our study is that we have to resort to published data from ICU cohorts, notably [3,4], to obtain a hypothesis about how earlier adequate treatment translates into reduced morbidity and mortality. Whether the differences observed between the included ICU cohorts were sufficiently balanced with respect to potential confounders, and whether they apply when we move about 40% of them (Figure 1, 40% = (10 + 19)/74) towards earlier adequate treatment, introduces uncertainty into our quantitative prediction.

We reported two independent predictions about the balance of incremental costs and effects from PCR testing. Taken in combination, cost-effective application will result with patients that are characterized by lower daily treatment cost, inadequate treatment rate and BC+ or PCR+ rate.

## Conclusions

Our analysis of observational data allows plausible predictions, characterizes patient groups of interest, and is balanced with respect to the sensitivity of results to key input. PCR detection promises to be cost-effective for improving antimicrobial treatment and medical outcome for septic post-surgical and ICU patients. However, prospective interventional studies are now needed to complement the insights regarding clinical benefit and cost-effectiveness of multiplex PCR-based diagnosis to improve adequacy of antimicrobial treatment.

## Key messages

• Multiplex PCR pathogen detection is useful as an adjunct to blood cultures to support early adjustment of empiric antimicrobial therapy.

• The incremental cost is justified for patients with over 25% inadequate initial treatment, especially in the presence of high daily treatment cost and risk of severe complications from inadequate treatment.

• The prediction model provides guidance when designing and evaluating interventional trials that incorporate PCR into managing antimicrobial treatment in sepsis. It can also be used to explore relative utility and price-worthiness of alternative molecular assays, or of how to best implement them into laboratory routines.

## Abbreviations

€: Euro (European currency unit); BC: blood culture (BC+: blood culture with clinically relevant microorganism identified, not counting contaminations that are immediately at reporting evident to the treating clinician); CI: confidence interval; CoNS: coagulase-negative *Staphylococcus*; Cost_surv_: total (PCR related, incremental) costs that are incurred per incremental survivor; ICER: incremental cost-effectiveness ratio; ICU: intensive care unit; LOS: length of stay (of a patient in a department, or in the hospital); MRSA: methicillin-resistant *Staphylococcus aureus*; N_epis_: total number of episodes (and tests) in which blood cultures are complemented by PCR testing for managing early antimicrobial treatment in sepsis; PCR: polymerase chain reaction (PCR+: PCR with clinically relevant microorganism identified. Note that in our study, all PCR+ reported microorganisms are considered clinically relevant); QALY: quality-adjusted life year.

## Competing interests

LEL, GJK and FS received research funding, reagents and equipment from Roche Diagnostics for the underlying project. BH is an employee of the manufacturer of the PCR assay. MHK declares that he has no competing interests.

## Authors' contributions

LEL has made substantial contributions to study conception and design, data collection and analysis, and wrote the manuscript. He was treating ICU physician in a participating site utilizing PCR. BH has made substantial contributions to study conception, developed the method section and did the calculations. Of note, the formulas and calculations were cross-checked, verified and supplemented with statistical calculations by HW Steinberg, Baseline GmbH (see Acknowledgments). GJK has made substantial contributions to study conception and design, data analysis, and strongly contributed to the study definitions and graphical content. MHK has made substantial contributions to study design, data acquisition and data analysis regarding outcomes associated with inadequate antibiosis in ICU sepsis. He contributed regarding tailoring content to the ICU readership. He is head of ICU in the participating site where the association between early inadequate treatment and outcomes was studied. FS has made substantial contributions to study conception, study design and data interpretation. He is head of ICU in a participating site where the PCR assay is implemented.

## Supplementary Material

Additional file 1 **Cost per PCR test**. This additional file explains the cost components for one PCR test. Furthermore, two graphs represent how the key results would change with different PCR costs. Click here for file

Additional file 2 **Substitution factors**. Description: For institutions that presently have only culture based data available, the substitutions in this additional data file allow utilization of the model to estimate potential gains from PCR. Click here for file
