# Supplementary material for: Cost and mortality prediction using polymerase chain reaction pathogen detection in sepsis: evidence from three observational trials
Source: Crit Care. 2010 Oct 15;14(5):R186. doi: 10.1186/cc9294 (PMC3219292; doi:10.1186/cc9294)

Additional data file 1

Cost per PCR test: Related laboratory cost components (A), and effect on break-even (B) respectively on cost per incremental survivor (C)

A

Cost component per test		Determination	- - - - - Variables and values - - - - - - -				Result [a]
			Samples per PCR run	PCR runs per year	Labora-tory invest	IT and initial training	
c_R	**Reagents and controls**	= (N(s/r) * 122 € + 201€) / N(s/r)	N(s/r) =4				**172 €**
c_L	**Labor** @ hourly cost c_{Lh}	= c_{Lh} * (N(s/r) *0.15hr +3 hr)/N(s/r)	N(s/r) =4				**29 €** @ c_{Lh} = 30 €
c_I	**Upfront invest**, t_Y years depreciated; f_S annual **maintain-ance** as % of invest	= (c_{IL}+c_{IT})/ (t_Y* N(r/yr) * N(s/r))+ (c_{IL}+c_{IT})*f_S/ (N(r/yr) * N(s/r))	N(s/r) =4	N(r/yr) =365*6/7	c_{IL} = 70000 €	c_{IT} = 18000 €	**21 €** @ t_Y = 5 yr f_S = 10%
c_O	**Overhead and risk**						**78 €**
Cost per PCR test		= c_R + c_L + c_I + c_O					**300 €**

[a] The Euro (€) cost (and any € results) can be transformed into other currencies by multiplying with the applicable exchange rate, for instance for US$ by multiplying with (1.50 $/€) or, if available, with (local $Cost_{/test}$ /300€).

B

Effect of PCR test cost on the resulting break even daily cost (Sav_{be})

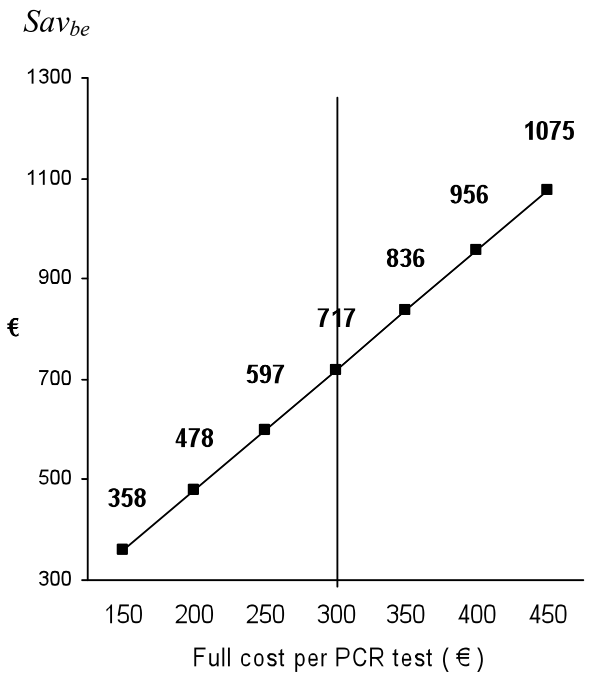

C

Effect of PCR test cost on the resulting cost per incremental survivor ($Cost_{surv}$)

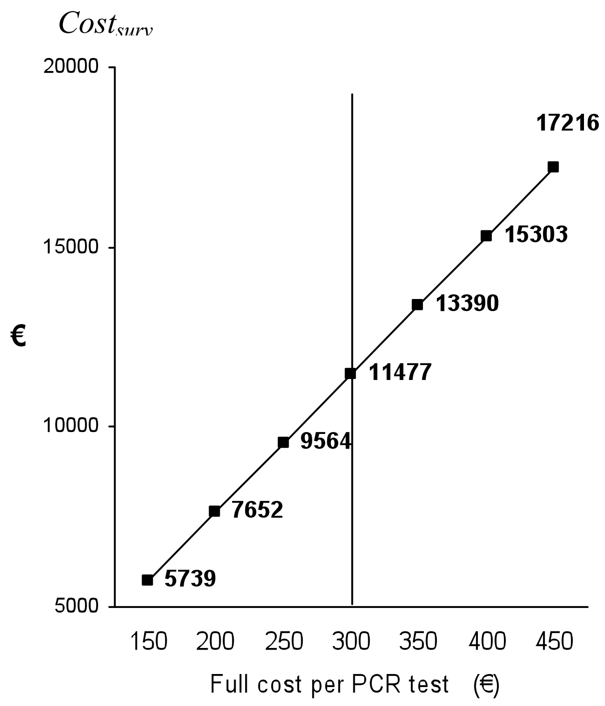

Supplement: Additional file 1 — Cost per PCR test. This additional file explains the cost components for one PCR test. Furthermore, two graphs represent how the key results would change with different PCR costs. [file cc9294-S1.PDF]
