# Supplementary material for: Cost and mortality prediction using polymerase chain reaction pathogen detection in sepsis: evidence from three observational trials
Source: Crit Care. 2010 Oct 15;14(5):R186. doi: 10.1186/cc9294 (PMC3219292; doi:10.1186/cc9294)
Supplement: Additional file 2 — Substitution factors. Description: For institutions that presently have only culture based data available, the substitutions in this additional data file allow utilization of the model to estimate potential gains from PCR. [file cc9294-S2.PDF]

Additional data file 2

Substitutions that allow predictions for patients without available PCR data

**Substitution** *for parameter :*	**in** equa-tion [ ]	**Substitution** [a]	**Source of cor-relation**	**Comment**
$Sh_{\ PCR+}$	*3, 4, 5*	$= Sh_{\ BC+} * 1.658$ [b]	Tab. 1	$Sh_{\ PCR+}$= 73/221=0,330;<br>$Sh_{\ BC+}$= 44/221=0,199
In_{PCR+}^{IA}	*3, 4, 5*	$= In_{total}^{\mathrm{mod}\ ific} * 1.185$	Fig. 1	$In_{total}^{\mathrm{mod}\ ific}$=74/221=0.335 (total adjustments)<br>In_{PCR+}^{IA} =29/73=0.397 (adjusted PCR+)
$M_{\ PCR+}^{\ IA}$	5	$= \dfrac{0{,}831 * M_{\ total}}{In_{total}^{\mathrm{mod}\ ific} + \dfrac{1 - In_{total}^{\mathrm{mod}\ ific}}{RR\dagger}}$	[3, 4]<br>Fig. 1	$RR\dagger$ =2.315 (relative risk of non-survival)<br>$M_{\ total}$ =59/189=0.312 (all patients);<br>$M_{\ PCR+}^{\ IA}$ = (9+3)/(19+10) =0.414;<br>$In_{total}^{\mathrm{mod}\ ific}$ =74/221= 0.335
In_{PCR+}^{IA}	*3, 4, 5*	$= In_{PCR+}^{\mathrm{mod}\ ific} * 0.829$	[17]	PCR+ had capacity to trigger 29 early ad-equate treatments along with 6 over-treat-ments => In interventional use, *35 PCR+ triggered modifications of treatment* would lead to the described effects, to which really 0.829*35= *29 inadequately treated* episodes contribute.

[a] **Definitions of substitution variables:**

Sh_{BC+}: Share of episodes for which at least one clinically significant microorganism was detected by blood culture.

$In_{total}^{\mathrm{mod}\ ific}$: Incidence of modification of initial antimicrobial treatment in the total patient cohort (between 12 hours and 7.5 days of initiation of first treatment or blood culture order), for coverage of different kinds of microorganisms or of different resistances.

$Mortality_{\ total}$: Mean mortality rate in the total patient cohort, within 30 days of discontinuing antimicrobial treatment

$In_{PCR+}^{\mathrm{mod}\ ific}$: Incidence of modification of initial antimicrobial treatment based on PCR+ results *in interventional use,* consisting of switches to earlier adequate treatment, and to over-treatment. The latter may not be easy to discern, even in retrospective analysis, once PCR+ results are utilized interventionally.

[b] The factor we use to estimate PCR+ share based on BC+ share describes the sensitivity advantage we saw in our ICU and post-surgical sepsis episodes when adding just *one* PCR test. Table 2 and, on a broader scale, reference [17] show possible variance with patient mix.
